# The Role of Accurate Estimations of Blood Loss and Identification of Risk Factors in the Management of Early Postpartum Hemorrhage in Women Undergoing a Cesarean Section

**DOI:** 10.3390/jcm14061861

**Published:** 2025-03-10

**Authors:** Zofia Włodarczyk, Aleksandra Śliwka, Hanna Maciocha, Szymon Paruszewski, Julia Wyszyńska, Maja Kłopecka, Gabriela Afrykańska, Marta Śliwińska, Artur Ludwin, Paweł Jan Stanirowski

**Affiliations:** 1st Department of Obstetrics and Gynecology, Medical University of Warsaw, 02-015 Warsaw, Poland

**Keywords:** postpartum hemorrhage, cesarean section, blood loss estimation, risk factors

## Abstract

**Objective:** This study aimed to analyze and compare three different methods of estimated blood loss (EBL) assessment in conjunction with the exploration of risk factors associated with early postpartum hemorrhage (PPH) among women undergoing a cesarean section (CS). **Methods:** Women with a singleton pregnancy who underwent an elective/emergency CS were recruited for this prospective cross-sectional study. Early PPH was defined as a cumulative blood loss ≥1000 mL within the 24 h period following the delivery. Methods of EBL assessment included the following: (1) visual estimation by the surgeon (sEBL), (2) the evaluation of blood-soaked dressings (dEBL), and (3) implementation of a mathematical formula (fEBL). **Results:** In the study period, 21 cases of early PPH were identified and compared with 452 controls. Among the patients with a PPH, a significant increase in the surgery time (60 min. vs. 46 min., *p* = 0.001), fetal birthweight (3780 g vs. 3417.5 g, *p* < 0.01), the occurrence of uterine atony (61.9% vs. 2.2%, *p* < 0.001), and myomas (9.5% vs. 1.1%, *p* < 0.05) was noted. In both groups, dEBL and sEBL provided the highest and the lowest EBL values, respectively (PPH dEBL: 1230 mL vs. fEBL: 1173.3 mL vs. sEBL 1000 mL, *p* < 0.001; control dEBL: 652 mL vs. fEBL 604 mL vs. sEBL 600 mL, *p* < 0.001). A patient age of 31–34 years (OR 1.71; 95%CI: 1.19–2.44), overweight (OR 2.65; 95%CI: 1.87–3.76), obesity (OR 2.68; 95%CI: 1.71–4.21), emergency mode of CS (OR 4.06; 95%CI: 2.94–5.62), surgeon experience (resident OR 1.86; 95%CI: 1.27–2.7; assistant specialist OR 3.13; 95%CI: 2.15–4.55) and fetal macrosomia (OR 3.19; 95%CI: 2.14–4.74) were selected as significant risk factors of the PPH. **Conclusions:** In women with early PPH following a CS, both dEBL and fEBL provide comparable estimations of blood loss. An emergency-mode CS and fetal macrosomia are the strongest contributors to PPH among women undergoing a CS. A combination of different methods of EBL with the proper identification of risk factors of a PPH can lead to improvement in the clinical management of obstetric hemorrhage following the CS.

## 1. Introduction

Despite continuous advancements in obstetric care, postpartum hemorrhage (PPH) remains a leading cause of maternal mortality worldwide. It is estimated that PPH and severe PPH account for ca. 6% and 1.9% of all deliveries, respectively [1]. It should be emphasized, however, that depending on the definition and the method used for blood loss estimation, the prevalence of PPH varies considerably between studies, which underscores the challenge of achieving consistency in its diagnosis [2].

A cesarean section (CS) is a commonly performed surgical procedure that carries the risk of multiple complications, both for the mother and the child. The recent data from 154 countries, representing 94.5% of global live births, indicate that 21.1% of women deliver via CS [3]. Considering the consistent upward trend in the number of surgical deliveries, a simultaneous increase in the rate of PPH should be anticipated. It is, therefore, important to accurately diagnose PPH and to implement preventive measures at an early stage, particularly in patients with risk factors for this complication.

Early PPH is defined as a cumulative blood loss of greater than or equal to 1000 mL or blood loss accompanied by signs and symptoms of hypovolemia within the 24 h period following the delivery [4]. As already mentioned, due to the differences in definitions and/or methods of blood loss evaluation, the reported prevalence of an early PPH varies significantly, ranging from 1.2% to 12.5% [5]. Given the rapid progression and the necessity for prompt intervention, the precise estimation of blood loss (EBL) is of the utmost importance for timely PPH management. Visual estimation by the surgeon—the most common method used in clinical practice—is subjective and often unreliable, usually leading to the underestimation of the actual blood loss [6,7,8]. In these circumstances, the implementation of critical interventions might be delayed. This limited accuracy of the visual estimation has prompted the exploration of alternative methods for quantifying blood loss, such as the evaluation of blood-soaked dressings or the utilization of mathematical formulas. The assessment of blood-soaked dressings can be conducted either by weighing them on a scale or by employing a visual evaluation. The former method involves weighing surgical dressings before and after use. This technique provides greater accuracy compared to visual estimation but requires additional resources and is time-consuming [5,9]. An alternative is the application of a visual assessment scale, which, while less precise, offers a faster and more straightforward approach, requiring only prior training to familiarize personnel with the method [10]. Finally, the mathematical evaluation of blood loss relies on measurable parameters, such as the difference in pre- and post-operative hematocrit levels; therefore, it has the advantage of being highly objective [5,9,11]. Nonetheless, this approach is based on theoretical assumptions, raising questions about its practical applicability and accuracy in real-world clinical settings. 

Achieving the effective control of PPH necessitates not only a reliable evaluation of blood loss but also a dedicated approach to prevention, which involves the prompt identification of high-risk patients and the implementation of a collaborative care model [12]. Several risk factors of PPH have been identified, including advanced maternal age, obesity, multiparity, history of a previous CS, uterine atony, multiple pregnancies, fetal macrosomia, or pre-eclampsia [13,14]. In addition, an emergency CS, often performed under less controlled circumstances, is associated with a higher risk of obstetric hemorrhage [15].

Taking into consideration the above-mentioned data, the main aim of the present study was to analyze and compare three different methods of blood loss estimation in women undergoing CS within a tertiary care facility in Poland. In addition, the evaluation of risk factors associated with an early PPH following a CS was performed as a part of a secondary analysis. By identifying high-risk patients and improving blood loss estimation techniques, this research intends to contribute to a reduction in maternal morbidity and mortality by optimizing clinical practices and, thus, improving outcomes in obstetric care.

## 2. Material and Methods

### 2.1. Study Design and Population

A prospective cross-sectional study was conducted between 1 January and 30 September 2023 in the 1 Department of Obstetrics and Gynecology, a tertiary referral center at the Medical University of Warsaw, performing approximately 1300 deliveries per year. The Local Ethics Committee approved the study (reference no. KB/36/2022 obtained on 14 March 2022), and written informed consent was obtained from all participants. Only patients who underwent singleton pregnancies with an elective/emergency CS and in whom all three methods of EBL were applied were found eligible for the study. To achieve methodological consistency and minimize the risk of bias, the research limited its analysis to surgical deliveries, acknowledging distinct bleeding patterns and the difficulties in EBL assessment following vaginal deliveries.

The technique of a CS, which encompasses a transverse skin incision (Pfannenstiel) followed by a transverse uterine incision in the lower segment, was used in all participants. Regarding PPH prevention, according to the recommendations of the Polish Society of Gynecologists and Obstetricians (PSGO), in women with known risk factors for uterine atony (e.g., multiple pregnancies, uterine myomas, polyhydramnios, history of ≥2 CS, fetal macrosomia, placenta previa, history of PPH or uterine atony) a prophylatic dose of carbetocin was administered after the delivery of the fetus [16].

Methods of EBL included the following: (1) visual estimation of blood loss by the surgeon (sEBL), (2) visual evaluation of blood-soaked surgical dressings (dEBLs), and (3) the application of a mathematical formula (fEBL). The study protocol did not incorporate the use of calibrated suction canisters during the CS as a method for EBL assessment, as this practice is not routinely adopted within the Department. Directly after the CS, sEBLs were applied by the surgeon performing the procedure based on personal experience. Subsequently, dEBLs were assessed according to the publication by Ali Algadiem et al. by the operating room (OR) nurse [10]. Briefly, in the study, the authors proposed the visual analog scale for EBL based on the size of commonly used gauze dressings (10 × 10 cm, 30 × 30 cm, and 45 × 45 cm) and their absorptive capacity. Twelve patterns representing increasing levels of blood saturation (25%, 50%, 75%, and 100%) were evaluated and pictured. Prior to the start of the presented study, all nurses were familiar with the method, and appropriate images of dressing sizes in conjunction with the percentage of saturation were made accessible in the OR. Only 10 × 10 cm and 45 × 45 cm gauze dressings were available for the CS during the study period. Following the surgery, all dressings used were evaluated, and the result from the dEBLs was inserted into the medical protocol. In order to prevent adjustments in the sEBL, the surgeon was not informed about the results of the dEBLs. Lastly, the fEBL method based on the formula accessible on the website was applied by the research team [17]. To enable mathematical evaluation of blood loss in all women, concentrations of erythrocytes, hemoglobin (Hgb), and hematocrit (Hct) were assessed prior to the delivery/CS and reevaluated 24 h after the surgical procedure. Metric units representing maternal weight and height were converted into imperial units: 1 pound = 2.2 × 1 kg and 1 inch = 39.37 × 1 m. The equations used for the fEBL calculation are given below:

1. Calculated pregnancy blood volume = 0.75 × ([maternal height (inches) × 50] + [maternal weight (pounds) × 25]);
2. Percentage of blood volume lost = (pre-delivery Hct − post-delivery Hct)/pre-delivery Hct;
3. fEBL = Calculated pregnancy blood volume x percentage of blood volume lost.

### 2.2. Study Outcome Definitions

An early PPH was defined as a cumulative blood loss of at least 1000 mL within the 24 h period following the CS [4]. Only cases in which EBL exceeded 1000 mL as assessed by each method (sEBL, dEBL, fEBL) were considered as a true PPH. The duration of the surgery was the time elapsed from the skin incision to closure measured by the OR nurse. An emergency CS was defined as a procedure performed within 30 min of the decision. The surgeon’s experience was determined on the basis of specialization in obstetrics and years of experience: resident—physician during specialist training; assistant specialist—specialist in obstetrics for ≤5 years; and consultant—specialist in obstetrics for >5 years. The night shift was a term used for a CS performed between 10:00 PM and 07:00 AM the following day. Indications for a CS were classified according to the recommendations of the PSGO [18]. Briefly, a first-stage arrest disorder was defined as a failure in the labor progress of women with ≥6 cm cervical dilation and at least 4 h of adequate uterine activity, and a second-stage arrest disorder was a failure in labor progress after 2 or 3 h of pushing in women without/with epidural anesthesia, respectively. Fetal malpresentation was classified as fetal presentation other than cephalic (breech, transverse) and cephalo-pelvic disproportion and was defined as the mismatch between the size of the fetal head and the maternal pelvis. Finally, fetal macrosomia was diagnosed when the fetal birthweight (FBW) exceeded 4000 g, irrespective of the gestational age.

### 2.3. Statistical Analysis

All calculations were performed using R software (R Core Team, Vienna, Austria), version 4.3.2. In the case of categorical data, the frequency distribution was presented, and the chi-square test or Fisher’s exact test was utilized to assess differences between the two groups. Continuous variables were compared using Student’s *t*-test or the Mann–Whitney U test following the distribution analysis. For comparisons of data from more than two groups, Friedman’s test was applied. Continuous data were expressed as the mean, standard deviation (SD), and range or as the median and interquartile range (IQR).

Associations between methods of blood loss estimation and selected maternal–fetal parameters were analyzed with the use of Spearman’s rank correlation coefficient (rho). Heatmaps were utilized to visualize correlations, employing both points and colors.

Finally, univariate and multivariate logistic models were constructed to test the relationships between selected maternal–fetal parameters and PPH occurrence. Based on the Akaike Information Criterion (AIC), the best-fit multivariate model was selected.

To our knowledge, no earlier research has concurrently examined similar approaches to blood loss evaluation within the obstetric setting, particularly regarding dEBLs; hence, a power analysis was not conducted, and the study results need to be considered preliminary.

The results were considered statistically significant if the *p*-value was <0.05.

## 3. Results

During the study period, there were 853 deliveries in the Department, including 536 CS (62.8%). Further exclusion of multiple pregnancies (74) resulted in 779 singleton deliveries comprising 473 CS (60.7%), which were subsequently qualified for the analysis. Following the completion of the study data, 21 cases of early PPH (21/473, 4.44%) were diagnosed and compared to 452 controls.

The study participant characteristics are presented in Table 1. There were no significant differences between both groups with regard to the majority of maternal data apart from a substantial increase in the surgery time (60 min. vs. 46 min., *p* = 0.001), the occurrence of uterine atony (61.9% vs. 2.2%, *p* < 0.001), and myomas (9.5% vs. 1.1%, *p* < 0.05) among women diagnosed with a PPH. Analysis of the laboratory results showed no significant differences with regard to the pre-delivery concentrations of erythrocytes, Hgb, and Hct. On the contrary, post-CS concentrations of erythrocytes (3.36 ± 0.28 mln/dL vs. 3.73 ± 0.34 mln/dL, *p* < 0.001), Hgb (10.3 ± 0.83 mg/dL vs. 11.2 ± 0.96 mg/dl, *p* < 0.001), and Hct (29.3 ± 2.2% vs. 32.8 ± 2.6%, *p* < 0.001) were significantly decreased in the study group. Correspondingly, significantly higher differences between the pre- and post-delivery levels of erythrocytes (0.79 mln/dL vs. 0.44 mln/dL, *p* < 0.001), Hgb (2.1 mg/dL vs. 1.2 mg/dL, *p* < 0.001), and Hct (7.4% vs. 3.8%, *p* < 0.001) were observed in women from the study group. There was only one case of blood transfusion in the PPH group and none in the control group. In this patient, fEBL was calculated based on the laboratory results obtained 6 h after the CS and prior to transfusion. Regarding neonatal outcomes, the only significant difference was the FBW. The neonates of mothers with an early PPH were significantly heavier compared to the controls (3780 g vs. 3417.5 g, *p* < 0.01). In addition, fetal macrosomia ≥4000 g occurred more frequently in the former group; however, the difference did not reach statistical significance (23.8% vs. 11.1%, *p* = 0.08).

The evaluation of EBL among women with a PPH and the controls showed that in both groups, dEBL and sEBL provided the highest and the lowest values, respectively (PPH dEBL: 1230 mL vs. fEBL: 1173.3 ml vs. sEBL 1000 mL, *p* < 0.001; control dEBL: 652 mL vs. fEBL 604 mL vs. sEBL 600 mL, *p* < 0.001), (Table 2). There were no significant differences between dEBL and fEBL values in the study groups. Conversely, in women from the control group, the dEBL value was significantly higher compared to the estimation based on the mathematical formula.

**Figure 1:**
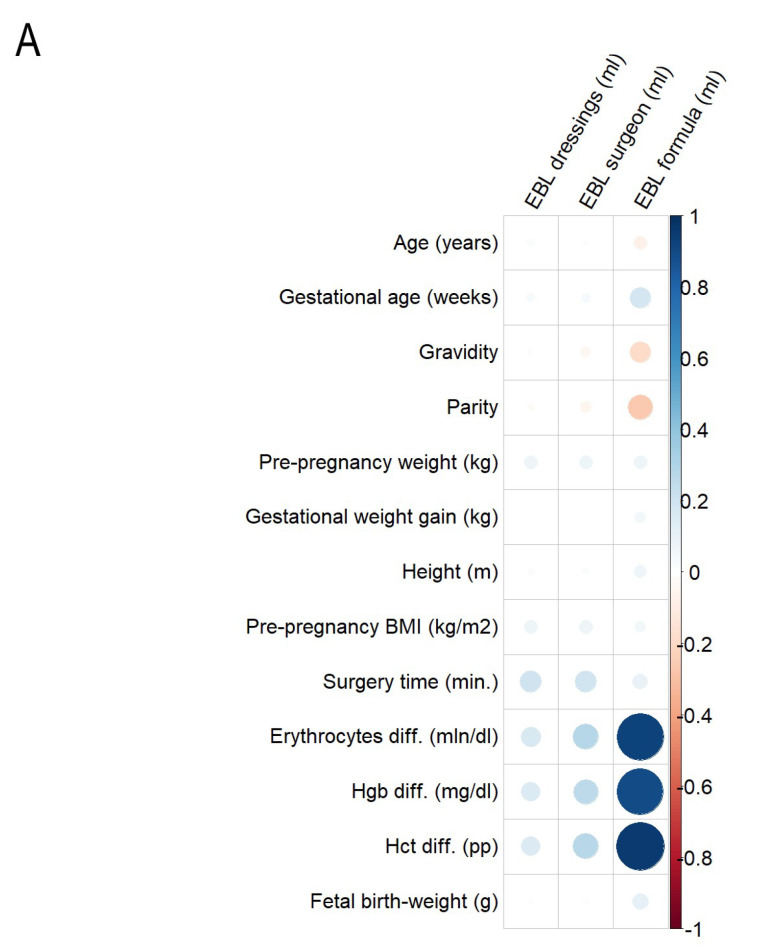

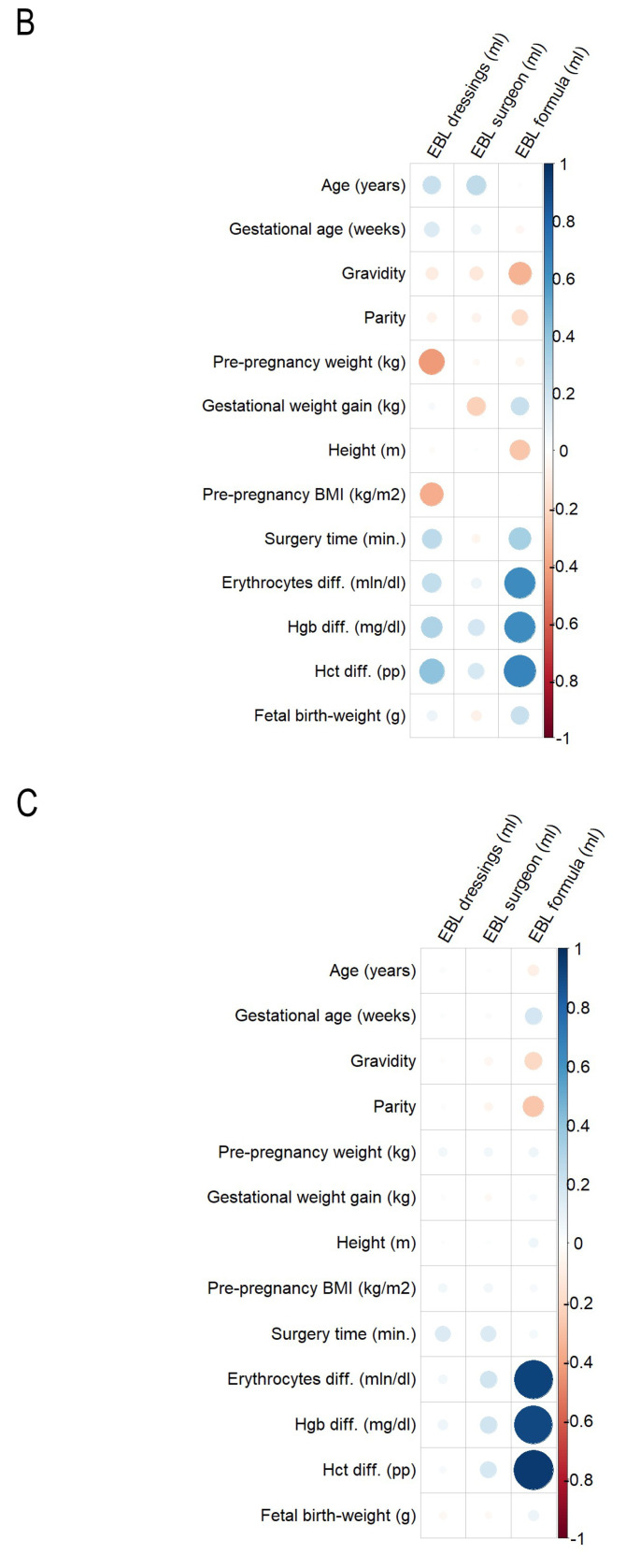
Heatmaps of correlations between the selected maternal–fetal parameters and methods of blood loss estimation ((**A**)—study + control group, (**B**)—study group, (**C**)—control group). BMI—body mass index; EBL—estimated blood loss; Hgb—hemoglobin; Hct—hematocrit. Heatmaps use colors and sizes to represent data values [smaller points represent weak correlations, while larger points indicate higher correlation strength; warmer colors (e.g., red, orange) indicate negative correlations, and cooler colors (blue shades) indicate positive correlations].

Correlation analysis performed in the total study population revealed the presence of positive associations between differences in the levels of erythrocytes, Hgb and Hct and dEBL (rho = 0.165; 0.155; 0.157, respectively, *p* < 0.01), sEBL (rho = 0.282; 0.264; 0.274, respectively, *p* < 0.01), and fEBL (rho = 0.928; 0.896; 0.959, respectively, *p* < 0.01), (Figure 1A). In addition, positive correlations between the surgery time and dEBL (rho = 0.202, *p* < 0.01), sEBL (rho = 0.197, *p* < 0.01), fEBL (rho = 0.099, *p* < 0.05), and FBW and fEBL (rho = 0.110, *p* < 0.05) and negative correlations between the gravidity and parity and fEBL (rho = −0.185 and—0.264, respectively, *p* < 0.01) were noted. In the separate sub-group analysis, moderate/strong and positive associations between differences in the levels of erythrocytes and Hgb, Hct, and fEBL in both groups were demonstrated (PPH group: rho = 0.622; 0.624; 0.661, respectively, *p* < 0.01; control group: rho = 0.923; 0.903; 0.954, respectively, *p* < 0.01), (Figure 1B,C). These were the only significant correlations observed among women with a PPH. On the contrary, in the control group, weak positive associations between differences in the levels of erythrocytes and Hgb, Hct, and sEBL (rho = 0.191; 0.192; 0.174, *p* < 0.01) as well as between the surgery time and dEBL and sEBL (rho = 0.160 and 0.156, respectively, *p* < 0.01) were present (Figure 1C). Lastly, gravidity and parity were negatively and weakly associated with fEBL (rho =−0.203 and−0.278, respectively, *p* < 0.01) among women without PPH (Figure 1C).

The independent contribution of selected factors to PPH occurrence was evaluated by univariate regression modeling (Table 3). The analysis revealed placenta previa (OR 4.16; 95%CI: 1.75–9.91, *p* = 0.001), placental abruption (OR 4.16; 95%CI: 1.75–9.91, *p* = 0.001), surgery time ≥60 min. (OR 4.1; 95%CI: 3.1–5.42, *p* < 0.001), second-stage arrest disorder (OR 2.77; 95%CI: 1.83–4.21, *p* < 0.001), fetal macrosomia (OR 2.47; 95%CI: 1.61–3.81, *p* < 0. 001), epidural anesthesia (OR 2.3; 95%CI: 1.54–3.36, *p* < 0.001), surgeon experience—assistant specialist (OR 2.21; 95%CI: 1.6–3.1, *p* < 0.001), emergency mode of CS (OR 2.05; 95%CI: 1.58–2.66, *p* < 0.001), and maternal overweight (OR 1.99; 95%CI: 1.47–2.69, *p* < 0.001) to be the factors that substantially increase the risk of an early PPH. On the contrary, factors such as pre-term delivery <37 weeks (OR 0.58; 95%CI: 0.34–0.99, *p* < 0.05), parity—2 (OR 0.57; 95%CI: 0.42–0.76, *p* < 0.001), gestational weight gain 10–14.99 kg (OR 0.64; 95%CI: 0.46–0.91, *p* < 0.01), night shift (OR 0.28; 95%CI: 0.17–0.45, *p* < 0.001) and FBW 3000–3499 g (OR 0.3; 95%CI: 0.2–0.47, *p* < 0.001) were associated with a decreased risk of obstetric hemorrhage.

In the multivariate regression analysis, patients aged 31–34 years (OR 1.71; 95%CI: 1.19–2.44, *p* < 0.01), overweight (OR 2.65; 95%CI: 1.87–3.76, *p* < 0.001), obesity (OR 2.68; 95%CI: 1.71–4.21, *p* < 0.001), emergency mode of CS (OR 4.06; 95%CI: 2.94–5.62, *p* < 0.001), surgeon experience—resident (OR 1.86; 95%CI: 1.27–2.7, *p* = 0.001); assistant specialist (OR 3.13; 95%CI: 2.15–4.55, *p* < 0.001), and fetal macrosomia (OR 3.19; 95%CI: 2.14–4.74, *p* < 0.001) were selected as the most significant risk factors of a PPH (Table 4). Similarly to the univariate model, performing a CS during the night shift significantly decreased the risk of hemorrhage (OR 0.1; 95%CI: 0.06–0.19, *p* < 0.001).

## 4. Discussion

The presented study was cross-sectional, aiming to evaluate the accuracy of three commonly used methods of blood loss estimation as well as to identify risk factors of early PPH among women undergoing a CS. The prevalence of early PPH during the study period amounted to 4.44%, which is similar to other authors’ observations [4]. The conducted analysis revealed that visual evaluation of the soaked surgical dressings and visual assessment by the surgeon provided the highest and the lowest values of the lost blood, respectively. In addition, the multivariate analysis of risk factors selected the emergency mode of CS and fetal macrosomia as the strongest contributors to early PPH.

Previously published studies attempted to ascertain the most accurate EBL method, as the visual evaluation of blood loss lacks objectivity and precision [7,8]. It has been suggested that one of the main reasons behind that inadequacy is the minimal training provided to improve this skill [6]. Interestingly, longer clinical experience or a higher degree of experience by the surgeon was not found to improve the above-mentioned assessment, and some studies have demonstrated that visual estimation performed by an anesthesiologist was more accurate compared to that conducted by an obstetrician [19,20]. It has been proven that sEBL generally underestimates blood loss compared to other methods, which may lead to the overlooking of PPH cases [11]. In accordance with those observations, our results show that in patients with significant blood loss—considered as an early PPH—the sEBL value was the lowest compared to other methods.

Currently, there are no data available to advocate that quantifying EBL through visual assessment of the dressings is more accurate than other methods; however, dEBL determines blood loss more carefully, as it is neither as subjective as sEBL nor as indirect as fEBL. We found dEBL to reach the highest values both in the study as well as in the control group; hence, our results indirectly align with the American College of Obstetricians and Gynecologists Committee Opinion stating that quantitative measurements are more accurate than visual estimation in determining blood loss during obstetric emergencies [21]. It should be noted, however, that in the current study, the dEBL method was based on an analog visual scale, while previously published studies have assessed blood loss exclusively by weighing the surgical pads pre- and post-operatively [9,10,19]. Contrary to the visual evaluation, weighing the dressings seems more accurate; however, it is a method that requires more time and resources. To the best of our knowledge, no research evaluating the clinical validity of the visual dEBL in the obstetric population has been conducted to date, which implies the need for further studies.

The mathematical method most often used for estimating blood loss during a CS comprises the formula that incorporates the difference in the hematocrit values before and after the surgery, along with the calculated maternal blood volume. Its main advantages include standardization, objectivity, and greater accuracy compared to methods based on visual estimation. On the other hand, a significant limitation of this approach is the delay in obtaining laboratory test results, as well as its retrospective nature, which makes the immediate initiation of treatment impossible. The method also does not account for individual physiological variations, including differences in the body’s hemodynamic response to blood loss or postpartum plasma volume fluctuations. Furthermore, some studies suggest that EBL based on the mathematical formula is significantly overestimated compared to actual blood loss [9,11]. In our study, dEBL and fEBL values were comparable among women with an early PPH, which indirectly excludes the possibility of overestimation. In addition, in the control population, no differences between the fEBL and sEBL were noted.

It is generally accepted that PPH prediction strategies should include risk factor evaluation before any childbirth. Maternal, fetal, as well as labor-associated risk factors should be taken into consideration. Based on the available data, uterine atony emerges as the main cause of PPH, with an estimated contribution of 60–80% to all cases [13,22]. Likewise, in our study, uterine atony was observed in 61.9% of patients with early PPH.

Regarding maternal factors, the mother’s age, as well as the pre-pregnancy BMI, contributed predominantly to PPH. With respect to the latter factor, overweight and obese women accounted for almost half of PPH cases. Moreover, women whose BMI exceeded 25 kg/m^2^ had an almost three times higher likelihood of experiencing early PPH compared to patients with normal BMI. These findings corroborate the research by Whitley et al., who also identified obesity as an independent risk factor for PPH, particularly among women undergoing a CS [23]. The observed increased risk of obstetric hemorrhage may be linked to factors such as the prolonged duration of surgery, difficulties during anesthesia, or altered uterine contractility in individuals with an elevated BMI [23].

In the previously published papers, patient aged <20 and ≥35 years were independently associated with PPH occurrence [5,24,25,26,27,28,29]. This bimodal risk pattern likely reflects underlying mechanisms, such as a low educational level and inadequate prenatal care in younger patients versus reduced uterine elasticity, placental disorders, and increased comorbidities in older women [29,30]. Surprisingly, in our population, only patients from the age group of 31–34 years experienced an increased risk of early PPH. Such findings may be attributed to the fact that this age group constituted the largest proportion of the current study population. It is, therefore, plausible that with a larger sample size, other age categories would emerge as significant risk factors for PPH, validating the conclusions of previously published studies.

Fetal macrosomia, defined as a fetal birthweight exceeding 4000 g, constitutes a well-established risk factor for PPH [26,31,32]. In accordance with the literature data in the present study, fetal macrosomia was significantly associated with early PPH. The median fetal birthweight in the PPH group was considerably higher than in the control group, and almost 24% of neonates of women who experienced obstetric hemorrhage weighed over 4000 g. These findings are consistent with Liu et al., who reported a significantly higher risk of severe PPH following deliveries of macrosomic fetuses [26]. The pathophysiological mechanism behind this association involves the increased risk of prolonged labor and overdistension of the uterus [26]. Both conditions are predisposing factors for uterine atony or birth trauma and, as a consequence, can compromise the ability of the uterus to contract and sustain effective hemostasis.

In concordance with our results, previous studies indicate that the emergency mode of a CS constitutes an independent risk factor for PPH compared to elective surgery [33,34]. This elevated risk is primarily attributed to unpredictable clinical circumstances, the urgency of the intervention, and the limited time available for thorough preparation of the patient and the surgical team. Moreover, emergency indications such as pre-eclampsia or placental abruption further contribute to the increased likelihood of uterine atony, which is a leading cause of PPH.

A surgeon’s experience plays a key role in minimizing the risk of CS complications, including PPH. Nonetheless, to the best of our knowledge, no studies on this topic have been conducted to date. We found a significantly increased risk of hemorrhage when the CS was performed by a physician during training or an obstetric specialist with less than 5 years of experience. In our setting, residents who had less experience tended to perform simpler procedures associated with a lower risk of PPH. In contrast, an assistant specialist often handles more complicated cases. Therefore, while the assistant specialist demonstrates advanced technical proficiency, the complexity of the surgeries performed could result in a higher risk of significant blood loss.

Previous reports indicate that CS performed at night is associated with greater blood loss, possibly due to the prolonged operative time, which serves as an independent risk factor for maternal morbidity [35]. In contrast, in the current study, performing surgery during the night shift significantly decreased the risk of PPH. Possible explanations for this observation are the fact that the most complicated cases of CS are scheduled during the day, and the majority of those occurring at night are performed by obstetric consultants.

The primary strength of the conducted study lies in its comprehensive comparison of three different methods for EBL, hence providing valuable insights into their accuracy and clinical applicability. Moreover, the recruitment of participants within a tertiary referral center ensured a wide spectrum of pregnancy pathologies as well as access to advanced medical care, which emphasizes the reliability and relevance of the findings. Nevertheless, the research has several limitations. One of them is the inclusion of a relatively small population of patients from a single medical center. In addition, the study population was further limited as only patients with singleton pregnancies with early PPH were included, and it is well documented that multiple pregnancies increase the risk of hemorrhage, as well as the fact that 0.2–0.8% of cases of PPH occur later than 24 h after delivery [5,36,37]. Future research, consisting of a more diverse patient group from various clinical settings, is, therefore, warranted. Finally, as we did not use suction canisters routinely during the CS, the actual blood loss could not be evaluated.

To conclude, the results of the presented study underscore the inaccuracy of the surgical estimation of blood loss during CS, with an even more profound measurement error in cases of PPH. This highlights the necessity of establishing a more reliable method of EBL. Visual assessment of surgical dressings appears to be a promising alternative, being both cost-effective and relatively easy to introduce. A combination of such methods with the proper identification of risk factors of PPH can lead to improvement in the clinical management of obstetric hemorrhage among women undergoing a CS.

## Figures and Tables

**Table 1:** Maternal and neonatal characteristics of the study population.

	PPH (n = 21)	Control Group (n = 452)	*p* Value
Age (years)	32	32	0.89
	[30–34]	[29–35]	
≤30	5 (23.8%)	122 (27%)	0.90
31–34	11 (52.4%)	192 (42.5%)	
35–39	4 (19%)	109 (24.1%)	
≥40	1 (4.8%)	29 (6.4%)	
Gestational age at delivery (weeks)	39	39	0.35
	[38–40]	[38–40]	
Pre-term delivery < 37 weeks	1 (4.8%)	36 (8%)	0.99
Gravidity	2	2	0.93
	[1–3]	[1–3]	
Parity	1	1	0.59
	[1–2]	[1–2]	
1	13 (61.9%)	239 (52.9%)	0.50
2	5 (23.8%)	162 (35.8%)	
≥3	3 (14.3%)	51 (11.3%)	
Pre-pregnancy weight (kg)	73	63	0.16
	[61–77.5]	[55–74.6]	
Gestational weight gain (kg)	15	12	0.15
	[11–18]	[9–16]	
<10	5 (23.8%)	120 (26.5%)	0.21
10–14.99	5 (23.8%)	186 (41.2%)	
15–19.99	7 (33.3%)	95 (21%)	
≥20	4 (19%)	51 (11.3%)	
Pre-delivery weight (kg)	82	77	0.06
	[77–91]	[68–87.2]	
Height (m)	1.67	1.65	0.52
	[1.64–1.69]	[1.62–1.7]	
Pre-pregnancy BMI (kg/m^2^)	23.8	22.8	0.23
	[21–27.3]	[20.4–26.6]	
<25	11 (52.4%)	297 (65.7%)	0.32
25–29.9	7 (33.3%)	95 (21%)	
≥30	3 (14.3%)	60 (13.3%)	
Mode of CS			
Elective	9 (42.9%)	274 (60.6%)	0.16
Emergency	12 (57.1%)	178 (39.4%)	
History of CS			
0	14 (66.7%)	279 (61.7%)	0.93
1	6 (28.6%)	142 (31.4%)	
≥2	1 (4.8%)	31 (6.9%)	
Indications for CS			
Intrauterine fetal asphyxia	3 (14.3%)	46 (10.2%)	0.17
1st-stage arrest disorder	4 (19%)	65 (14.4%)	
2nd-stage arrest disorder	4 (19%)	42 (9.3%)	
Malpresentation	0 (0%)	37 (8.2%)	
Cephalo-pelvic disproportion	0 (0%)	15 (3.3%)	
Placenta previa	1 (4.8%)	7 (1.5%)	
Placental abruption	1 (4.8%)	7 (1.5%)	
Other	8 (38.1%)	233 (51.5%)	
Pre-eclampsia	0 (0%)	10 (2.2%)	0.99
Myomas	2 (9.5%)	5 (1.1%)	**0.03**
Duration of surgery (min.)	60	46	**0.001**
	[50–70]	[39–58]	
<30	0 (0%)	17 (3.8%)	**<0.01**
30–59.9	9 (42.9%)	328 (72.6%)	
≥60	12 (57.1%)	107 (23.7%)	
Night shift	1 (4.8%)	69 (15.3%)	0.34
Type of anesthesia			
Spinal	17 (81%)	397 (87.8%)	0.28
Epidural	4 (19%)	41 (9.1%)	
General	0 (0%)	14 (3.1%)	
Surgeon experience			
Resident	7 (33.3%)	171 (37.8%)	0.34
Assistant specialist	9 (42.9%)	126 (27.9%)	
Consultant	5 (23.8%)	155 (34.3%)	
Uterine atony	13 (61.9%)	10 (2.2%)	**<0.001**
Erythrocytes concentration before CS (mln/dL)	4.21 ± 0.37	4.16 ± 0.34	0.58
	(3.47–4.95)	(2.83–5.16)	
Erythrocytes after CS (mln/dL)	3.36 ± 0.28	3.73 ± 0.34	**<0.001**
	(2.92–4.03)	(2.54–4.79)	
Erythrocytes diff. (mln/dL)	0.79	0.44	**<0.001**
	[0.7–0.93]	[0.28–0.58]	
<0.7	5 (23.8%)	402 (88.9%	**<0.001**
≥0.7	16 (76.2%)	50 (11.1%)	
Hgb concentration before CS (mg/dL)	12.6 ± 1.24	12.4 ± 1	0.48
	(9.9–14.8)	(8.8–15.3)	
Hgb concentration after CS (mg/dL)	10.3 ± 0.83 (8.6–12.1)	11.2 ± 0.96 (8–14.4)	**<0.001**
Hgb diff. (mg/dL)	2.1	1.2	**<0.001**
	[1.9–2.9]	[0.8–1.7]	
<2	6 (28.6%)	394 (87.2%)	**<0.001**
≥2	15 (71.4%)	58 (12.8%)	
Hct before CS (%)	37.2 ± 3	36.6 ± 2.6	0.41
	(30.9–42.8)	(25.6–44.1)	
Hct after CS (%)	29.3 ± 2.2	32.8 ± 2.6	**<0.001**
	(25.5–33.7)	(23–42.1)	
Hct diff. (%)	7.4	3.8	**<0.001**
	[6.8–8.2]	[2.5–5.1]	
<7	7 (33.3%)	431 (95.4%)	**<0.001**
≥7	14 (66.7%)	21 (4.6%)	
FBW (g)	3780	3417.5	**<0.01**
	[3480–3950]	[3060–3747.5]	
<3000	4 (19%)	99 (21.9%)	**<0.05**
3000–3499	2 (9.5%)	163 (36.1%)	
3500–3999	10 (47.6%)	140 (31%)	
≥4000	5 (23.8%)	50 (11.1%)	
Fetal macrosomia ≥ 4000 g	5 (23.8%)	50 (11.1%)	0.08
Fetal sex			
Male	11 (52.4%)	231 (51.1%)	0.99
Female	10 (47.6%)	221 (48.9%)	
1st minute Apgar	10	10	0.63
	[10-10]	[10-10]	
5th minute Apgar	10	10	0.64
	[10-10]	[10-10]	
10th minute Apgar	10	10	0.73
	[10-10]	[10-10]	
Umbilical artery pH	7.3	7.29	0.50
	[7.28–7.34]	[7.26–7.32]	

Data are expressed as mean ± SD/(range), median and [IQR] or as frequency (%). BMI—body mass index; CS—cesarean section; FBW—fetal birthweight; PPH—postpartum hemorrhage; erythrocytes diff.—difference between pre- and post-operative concentrations of erythrocytes; Hgb—hemoglobin concentration; Hgb diff.—difference between pre- and post-operative hemoglobin levels; Hct—hematocrit; Hct diff.—difference between pre- and post-operative hematocrit; SD—standard deviation; IQR—interquartile range.

**Table 2:** Estimated blood loss according to the type of method applied.

Group	dEBL (mL)	sEBL (mL)	fEBL (mL)	*p* Value
PPH (n = 21)	1230	1000	1173.3	**<0.001 ^a^**
	[1230–1288]	[1000–1000]	[1126.6–1351.8]	**<0.05 ^b^**
Control group (n = 452)	652	600	604	**<0.001 ^a,c^**
	[652–778]	[500–700]	[379.8–792.3]	

Data are expressed as median and [IQR]. PPH—postpartum hemorrhage; dEBL—dressing-estimated blood loss; sEBL—surgeon-estimated blood loss; fEBL—formula-estimated blood loss; IQR—interquartile range. ^a^—dEBL vs. sEBL. ^b^—fEBL vs. sEBL. ^c^—dEBL vs. fEBL.

**Table 3:** Univariate analysis of risk factors of early postpartum hemorrhage among women undergoing cesarean section *.

Risk Factor	Estimate	OR	95%CI	*p* Value
Age (years)				
31–34	0.335	1.4	1.02–1.91	**<0.05**
35–39	−0.110	0.9	0.61–1.3	0.56
≥40	−0.173	0.84	0.46–1.53	0.57
Pre-term delivery < 37 weeks	−0.549	0.58	0.34–0.99	**<0.05**
Parity				
2	−0.567	0.57	0.42–0.76	**<0.001**
≥3	0.078	1.08	0.73–1.6	0.70
Gestational weight gain (kg)				
10–14.99	−0.438	0.64	0.46–0.91	**<0.01**
15–19.99	0.57	1.77	1.24–2.53	**<0.01**
≥20	0.63	1.88	1.23–2.9	**<0.01**
Pre-pregnancy BMI (kg/m^2^)				
25–29.9	0.688	1.99	1.47–2.69	**<0.001**
≥30	0.3	1.35	0.92–1.98	0.12
Emergency CS	0.719	2.05	1.58–2.66	**<0.001**
History of CS				
1	−0.172	0.84	0.63–1.12	0.23
≥2	−0.442	0.64	0.37–1.13	0.12
Indication for CS				
Intrauterine fetal asphyxia	0.642	1.9	1.24–2.91	**<0.01**
1st-stage arrest disorder	0.584	1.8	1.23–2.62	**<0.01**
2nd-stage arrest disorder	1.02	2.77	1.83–4.21	**<0.001**
Placenta previa	1.43	4.16	1.75–9.91	**0.001**
Placetnal abruption	1.43	4.16	1.75–9.91	**0.001**
Surgery time (min.)				
≥60	1.41	4.1	3.1–5.42	**<0.001**
Night shift	−1.3	0.28	0.17–0.45	**<0.001**
Epidural anesthesia	0.82	2.3	1.54–3.36	**<0.001**
Surgeon experience				
Resident	0.238	1.27	0.92–1.75	0.15
Assistant specialist	0.795	2.21	1.6–3.1	**<0.001**
FBW (g)				
3000–3499	−1.19	0.3	0.2–0.47	**<0.001**
3500–3999	0.57	1.77	1.24–2.51	**0.001**
≥4000	0.91	2.47	1.61–3.81	**<0.001**

*—due to significant disproportions between both groups, factors such as myomas (OR 9.41; 95%CI: 3.77–23.5, *p* < 0.001) and uterine atony (OR 71.8; 95%CI: 37.9–136, *p* < 0.001) were not included in the analysis. BMI—body mass index; CS—cesarean section; FBW—fetal birthweight; OR—odds ratio; CI—confidence interval.

**Table 4:** Risk factors associated with early postpartum hemorrhage following cesarean section: results from the multivariate analysis.

Risk Factor	Estimate	OR	95%CI	*p* Value
Age (years)				
31–34	0.535	1.71	1.19–2.44	**<0.01**
35–39	−0.188	0.83	0.54–1.27	0.39
≥40	0.082	1.09	0.56–2.08	0.8
Pre-pregnancy BMI (kg/m^2^)				
25–29.9	0.975	2.65	1.87–3.76	**<0.001**
≥30	0.987	2.68	1.71–4.21	**<0.001**
Emergency CS	1.402	4.06	2.94–5.62	**<0.001**
Night shift	−2.272	0.10	0.06–0.19	**<0.001**
Surgeon experience				
Resident	0.618	1.86	1.27–2.7	**0.001**
Assistant specialist	1.141	3.13	2.15–4.55	**<0.001**
Fetal macrosomia ≥ 4000 g	1.159	3.19	2.14–4.74	**<0.001**

BMI—body mass index; CS—cesarean section; OR—odds ratio; CI—confidence interval.

## Data Availability

The datasets used and/or analyzed during the current study are available from the corresponding author upon reasonable request.
